# Immune Checkpoint Molecules in Reproductive Immunology

**DOI:** 10.3389/fimmu.2019.00846

**Published:** 2019-04-18

**Authors:** Eva Miko, Matyas Meggyes, Katalin Doba, Aliz Barakonyi, Laszlo Szereday

**Affiliations:** ^1^Department of Medical Microbiology and Immunology, Medical School, University of Pécs, Pécs, Hungary; ^2^Janos Szentagothai Research Centre, Pécs, Hungary

**Keywords:** immune checkpoint molecule, reproductive immunology, pregnancy, immunotolerance, CTLA-4, TIM-3, PD-1

## Abstract

Immune checkpoint molecules, like CTLA-4, TIM-3, PD-1, are negative regulators of immune responses to avoid immune injury. Checkpoint regulators are thought to actively participate in the immune defense of infections, prevention of autoimmunity, transplantation, and tumor immune evasion. Maternal-fetal immunotolerance represents a real immunological challenge for the immune system of the mother: beside acceptance of the semiallogeneic fetus, the maternal immune system has to be prepared for immune defense mostly against infections. In this particular situation, the role of immune checkpoint molecules could be of special interest. In this review, we describe current knowledge on the role of immune checkpoint molecules in reproductive immunology.

## Introduction

The activation of the immune system to eliminate harmful agents is usually followed by tissue damage at the site of the exposure. In order to keep this side effect of the immune response limited and localized, efficient immunoactivation of immune cells requires multiple incoming signals. Beside antigen recognition, co-stimulatory, survival, and proliferative signals, even environmental factors can determine the outcome of the immune response (1–4).

Immune checkpoint molecules are co-stimulatory receptors, occurring on the surface of several immune cells. After ligand binding, these regulators are capable of transducing inhibitory signals (5). CTLA-4, TIM-3, PD-1 are the most studied members from this group of cell surface receptors (5). The physiological role of immune checkpoints is to prevent a harmful immune attack against self-antigens during an immune response by negatively regulating the effector immune cells, e.g., by inducing T cell exhaustion (5, 6). Recent studies suggest that each checkpoint decreases immunoactivation through different intracellular signaling mechanisms (5, 7). Immune checkpoint regulators are thought to actively participate in the immune defense of infections, prevention of autoimmunity, transplantation, and tumor immune evasion (5, 7).

Pregnancy is a natural model of active immunotolerance, where maternal immune system simultaneously faces two challenges: beside acceptance of the semiallogeneic fetus, the maternal immune system has to be prepared for immune defense mostly against infections. In this particular situation, the role of immune checkpoint molecules could be of special interest. Therefore, this paper aims to review the literature presenting current knowledge about the role of immune checkpoint molecules in reproductive immunology.

## CTLA-4

The first described inhibitory receptor CTLA-4 (Cytotoxic T-lymphocyte-associated protein 4) is predominantly and constitutively expressed intracellularly in regulatory T cells, and it is missing in naive conventional T cells (8, 9). Following activation, CTLA-4 is expressed on the cell surface of Tregs, but it can also be found on the cell surface of activated CD8+ or CD4+ T cells (10). The inhibitory effect of CTLA-4 results from the competition with the T cell activatory CD28 receptor to bind the B7 ligands CD80/CD86 present on the cell surface of antigen presenting cells (11). The ability of Treg cells to induce IDO expression in APCs through the CTLA-4-B7 binding was thought to be one of the major mechanism of immune suppression by these cells (12, 13). Interestingly, current thinking suggests, that the main function of CTLA-4 is not delivering negative signals through ligand binding but the removal of its ligands CD80/CD86 from the cell surface of APCs preventing thereby their binding to the costimulatory CD28 present on T cells (8, 14).

### CTLA-4 in Murine Pregnancy

The significance of the CD80/86-CD28 activation pathway in T cells during fetal rejection was shown by blocking both ligands with mAb during the pregnancy of the abortion-prone murine model. The blockade resulted in the improvement of fetal survival with an increase of Th2 type cytokines at the maternal-fetal interface (MFI) and in the peripheral expansion of the CD4+ C25+ T cell population. Furthermore, CTLA-4 expression by T cells increased as well which was found to be significantly reduced at the MFI in abortion-prone matings (15, 16). Preventing binding of CD80/CD86 to CD28 is thought to be the way of action of CTLA-4 with similar beneficial effects in maternal-fetal tolerance. Blocking only CD86 using the same experimental setting resulted in the same observations (17). These findings support previous theories about CD80 might be the most functional ligand for CTLA-4. Blockade of the CD86 could turn off the co-stimulatory CD86/CD28 pathway while allowing a prolonged CD80/CTLA-4 interaction with all of the benefits (17–19).

Further evidence for the immunosuppressive capacity of CTLA-4 was delivered from experiments with the CTLA4Ig fusion protein. Using an adenoviral vector, CTLA4Ig was shown to be heavily expressed at the MFI. CTLA4Ig therapy of abortion-prone CBA/DBA matings could effectively improve pregnancy outcome by shifting serum cytokine levels toward Th2 bias and expanding regulatory T cell population at the periphery (20). Furthermore, the CTLA4Ig fusion protein significantly inhibited splenic lymphocyte proliferation and apoptosis of the fetoplacental unit (21). Interestingly, adoptive transfer of Treg with CTLA-4 blockade from normal pregnant mouse to CBA/DBA pregnancy didn't abolish the protective effect of Treg treatment without a blockade resulting in decreased abortion rates (22).

In another abortion-prone setting, in sonic stressed pregnant mice, decidual lymphocytes expressed decreased levels of CTLA-4, without any changes in CD28 expression suggesting the failure of the control of local immunoactivation. CTLA-4 expression by decidual lymphocytes of stressed animals could be enhanced by injections of the dipeptidyl peptidase IV inhibitor, a well-known terminator of T-cell activation (23, 24).

### CTLA-4 in Human Pregnancy (Figure 1)

#### CTLA-4 at the Periphery

Although regulatory T cells increase in number in the periphery during early pregnancy, the enhanced CTLA-4 expression on the cell surface was not observed (10, 25). In contrast to these findings, the expression of one of the ligands of CTLA-4, namely CD86 showed an increased expression by peripheral DCs and monocytes in healthy pregnancy while CD80 expression patterns did not change (26).

**Figure 1:**
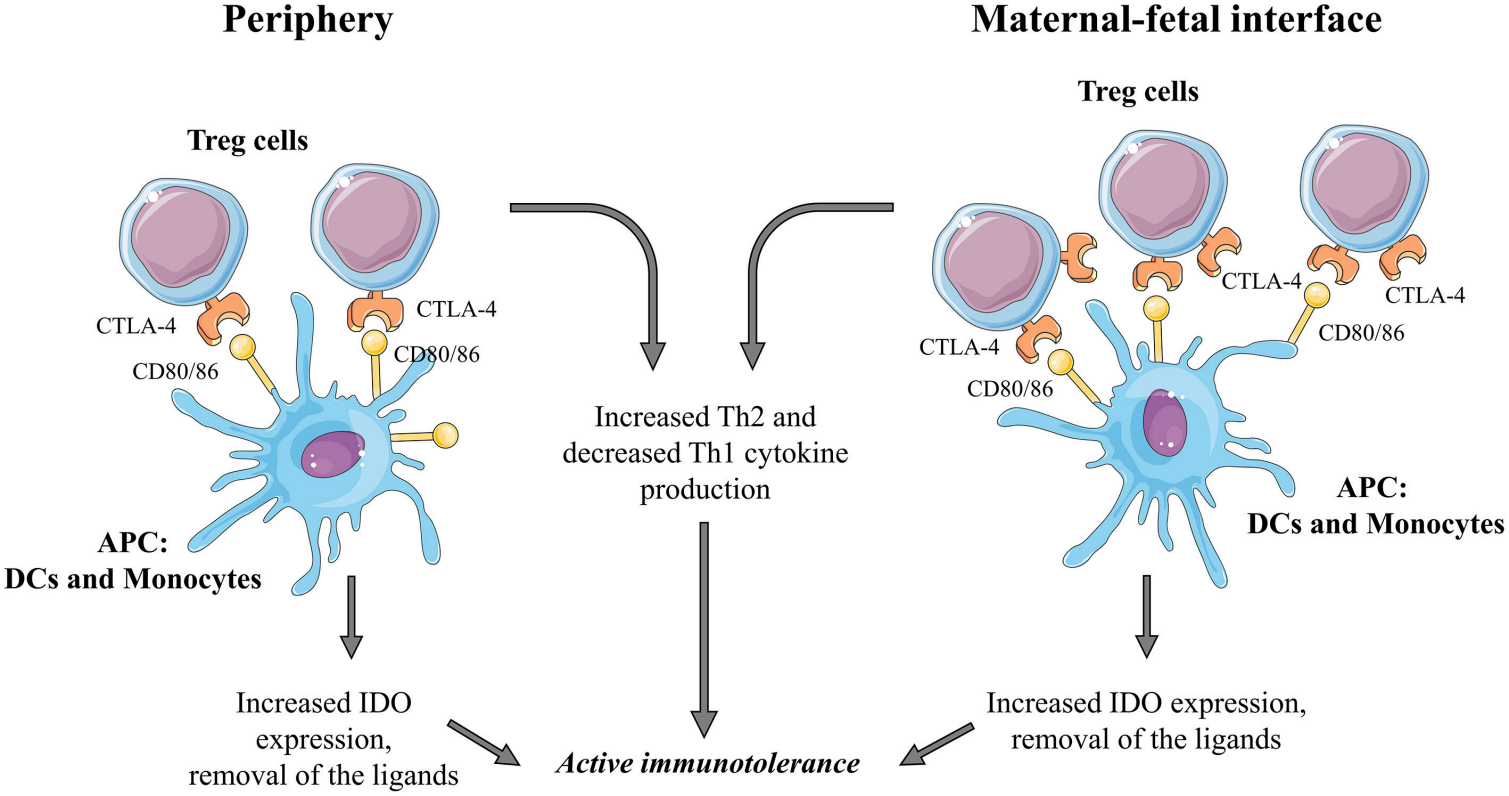
Summarizing the possible role of the CTLA-4/CD80, CD86 pathway during human pregnancy.

CTLA-4Ig treatment of peripheral blood mononuclear cells resulted in a significantly higher IFN-γ secretion in normal pregnancy compared to non-pregnant condition (26). Despite the fact, that CTLA-4 is capable of inducing indoleamine 2,3 dioxygenase (IDO) expression in dendritic cells and monocytes through the induction of IFN-γ, there are conflicting data about whether CTLA-4Ig treatment could enhance IDO expression in DCs and monocytes in normal pregnancy (13, 26, 27).

#### CTLA-4 at the Maternal-Fetal Interface

Compared to the periphery, decidual Treg cells further increase in number, and the frequency of Treg expressing intracellular or surface CTLA-4 was also found to be elevated in the decidua (10, 27, 28). Interestingly, placental fibroblasts also express CTLA-4, but it is supposed to have non-immunological functions since fibroblasts are not directly in contact with maternal tissues (29). The CTLA-4 ligands, CD80, and CD86, are also present on decidual DCs and monocytes, and they show the same expression profile as in the periphery in normal pregnancy (26, 30). The decidual CTLA-4 expression is in a significantly positive correlation with decidual Th2 cytokine production and a negative correlation with decidual Th1 cytokine production suggesting remarkable immunosuppressive effects locally (30).

CTLA-4Ig treatment of decidual lymphocytes resulted in enhanced IFN-γ and IDO expression (10).

#### CTLA-4 in Pregnancy Complications

In the case of spontaneous abortion/miscarriage peripheral and decidual Tregs fail to increase to the levels observed in normal pregnancy (10). Data about CTLA-4 expression in these conditions are conflicting. In one hand, the overall ratio of CTLA-4+ peripheral and decidual lymphocytes as wells as the ratio of CTLA-4+ Tregs was found to be significantly reduced. Moreover, the ratios of CTLA-4+/CD28+ in regulatory T cells from miscarriage were significantly lower than that of normal pregnancy (30, 31). On the other hand, there was no significant difference in intracellular and cell surface expression of CTLA-4 on both peripheral and decidual Tregs when compared to non-pregnant and healthy pregnant controls (10). These controversy data may result from different patient inclusion criteria. From the two possible CTLA-4 ligands, only CD86 expression was found to be affected in miscarriage: peripheral monocytes, decidual monocytes, and DC showed significantly lower expression rates compared to those in normal pregnancy (26, 30). Response levels of IDO expression by both peripheral and decidual monocytes and DCs in spontaneous abortion with CTLA-4 treatments were lower compared to a healthy pregnancy (26).

Extensive research focused on the role of *CTLA-4* gene polymorphism with different conclusions (32–37). The A/G polymorphism at position 49 in exon 1 of cytotoxic T lymphocyte antigen-4 (*CTLA-4*) gene may result in abnormal protein modification in the rough endoplasmic reticulum leading to reduced expression (38, 39). Further studies confirmed, that the 49 GG genotype was associated with a reduced inhibitory function of CTLA-4 whereas individuals with AA genotype had more expression of CTLA-4 both intracellular as on the cell surface of activated T cells (33, 40, 41). Further studies with larger sample sizes are needed to prove increased frequencies of G allele and GG genotype among patients with recurrent miscarriage.

Although preeclampsia is characterized by a diminished Treg frequency, a well-known alteration (42–46), little information is available about the possible role of CTLA-4 in the pathogenesis of the disease. CD80 and CD86 ligand expression levels on monocytes decrease in preeclampsia, while data about CTLA-4 expression of Treg are not conclusive, increased and unchanged expression patterns were reported as well. Therefore, it is difficult to determine the significance of the CTLA-4 pathway in preeclampsia (47–49). Two gene polymorphism studies of the exon-1 A49G region of the *CTLA-4* gene revealed an increased frequency of the heterozygosity and GG phenotype in pre-eclamptic women (38, 50).

In women with successful IVF treatment, there is an increase in the peripheral Treg population compared to failed IVF attempts. Investigating CTLA-4 expression at the mRNA level, no differences could be observed in the two IVF patient group (51).

Heterozygous mutations in the immune checkpoint protein CTLA-4 leading to CTLA-4 deficiency results in different autoimmune clinical features, but no further information is available about pregnancy proceeding in these patients (52, 53).

## TIM-3

Extensive research has established that Tim-3/gal-9 pathway plays a significant role in the regulation of immune responses and induction of tolerance (54–58). TIM-3 was shown to be expressed by many types of immune cells, including Th1, Th17, NK and NKT-like cells, Tregs, and also on antigen-presenting immune cells (59). Interestingly, TIM-3 activity is thought to participate in both activation and inhibition of immune response (60, 61). In the case of a healthy pregnancy, expression of TIM-3 on Th1 cells may be a key element for reducing proinflammatory Th1-dependent T-cell response (57).

The ligand of TIM-3 receptor is galectin-9 (Gal-9), a β-galactose binding protein (62). Among other identified receptors of Gal-9, TIM-3 has been studied most intensively (54). Both in mice and humans, binding of TIM-3 to its ligand Gal-9 leads to the apoptosis of Th1 and Th17 cells and induce immunotolerance (63–65). Thus, the TIM-3/Gal-9 pathway may serve as a checkpoint regulator limiting the Th1- and Th17-driven immune response and modulating the Th1/Th2 cytokine balance (54).

### TIM-3 in Murine Pregnancy

TIM-3 has been studied in detail in murine pregnancy models by several groups (66–71). First, immunofluorescence stainings revealed the presence of TIM-3 in midgestational uterus and flow cytometric analysis proved that this inhibitory molecule is expressed by a variety of immune cells residing locally in the uterus/decidua: uterine NK cells, γ/δ T cells, NKT-like cells, macrophages, dendritic cells (DC), and even by myeloid-derived (66–68). TIM-3 expression by these cells was shown to be dominant but variable throughout pregnancy, in the case of the most prevalent decidual immune cell type, NK cells upregulate TIM-3 during the first half of murine gestation (66, 67). Although TIM-3 expression of decidual NK cells and γ/δ T cells is similar to that in the periphery, their upregulated relative TIM-3 expression locally suggest that these cells are more mature and entirely functional (68, 72). However, the cytotoxic capacity of TIM-3+ decidual NK cells and γ/δ T cells was shown to be reduced when compared to the periphery; this might be due to the special local microenvironment at the MFI (68). In contrast to these findings, there is a smaller TIM-3+ NKT-like cell subset in the decidua with stronger lytic capacity. Therefore, separate action of TIM-3 on different immune cell types with varying functional outcomes could be concluded (68).

The TIM-3 ligand, galectin-9 is also present at the MFI at different sites. Both murine placental spongiotrophoblast and decidual regulatory T cells express galectin-9 and decidual Gal-9+ Th cells are the main source of the secreted, soluble form of Gal-9 (68). Since the presence of both the ligand, Gal-9 and its receptor, TIM-3 side by side, their binding interaction could be hypothesized, and the inhibitory signal derived from TIM-3 might contribute to maternal immunotolerance observed in murine pregnancy. This hypothesis is supported by the observation that TIM-3 blockade of allogeneic murine pregnancy resulted in litter size reduction, reduced live births, and an increased rate of resorption *in vivo* (66, 71).

Blocking TIM-3 with monoclonal antibodies (mAbs) provided further information about the possible function of this molecule at the MFI. Following inhibition, both apoptotic cells and macrophages accumulate locally, suggesting a deficiency of phagocytic clearance via failed recognition of phosphatidylserine through TIM-3 and enhanced pro-inflammatory cytokine production (66). Uterine granulocytes were also shown to increase in number and to enhance Th1 cytokine expression. These observations are in line with previous studies of experimental autoimmune/ischemic murine models where increased inflammation was due to macrophage and granulocyte activation following TIM-3 blockade (73, 74). Blocking TIM-3 on uterinal NK (uNK) cells affect both physiologic phenotype and function of these dominant cell population at the MFI (67). Although local accumulation and cytotoxic capacity of TIM-3+ uNK cells did not change, uNK cells upregulated the activation marker CD69, and their expression pattern of activating and inhibitory cell surface receptors was notably altered. Secretion of both proangiogenic (VEGF, IFN-γ) and immunosuppressive (IL-10) cytokines by TIM-3+ uNK cells were decreased. Additionally, TIM-3 inhibition resulted in reduced placental expression of the cytokines IL-15 and IL-9, which are important factors for NK cell survival and development (67, 75).

In abortion-prone mouse models, a reduced number of TIM-3+ dNK and CD4+ Th cells can be observed with predominantly Th1 cytokine profiles (69, 70).

All these data from murine pregnancy models suggest a protective role of TIM-3 present at the MFI.

### TIM-3 in Human Pregnancy (Figure 2)

#### TIM-3 at the Periphery

In pregnant women, upregulation of TIM-3 expression by peripheral leukocytes throughout pregnancy was mainly observed on monocytes and NK cells (59, 76). The percentage of TIM-3+ Th, Tc, and NKT-like cells remained relatively constant (57). In the third trimester of a healthy pregnancy, among lymphocytes, ~80% of NK cells, 15% of CD8+ T cells express TIM-3, in the case of CD4+ T, and NKT-like cells, the ratio of TIM3+ cells was below 5% (77).

**Figure 2:**
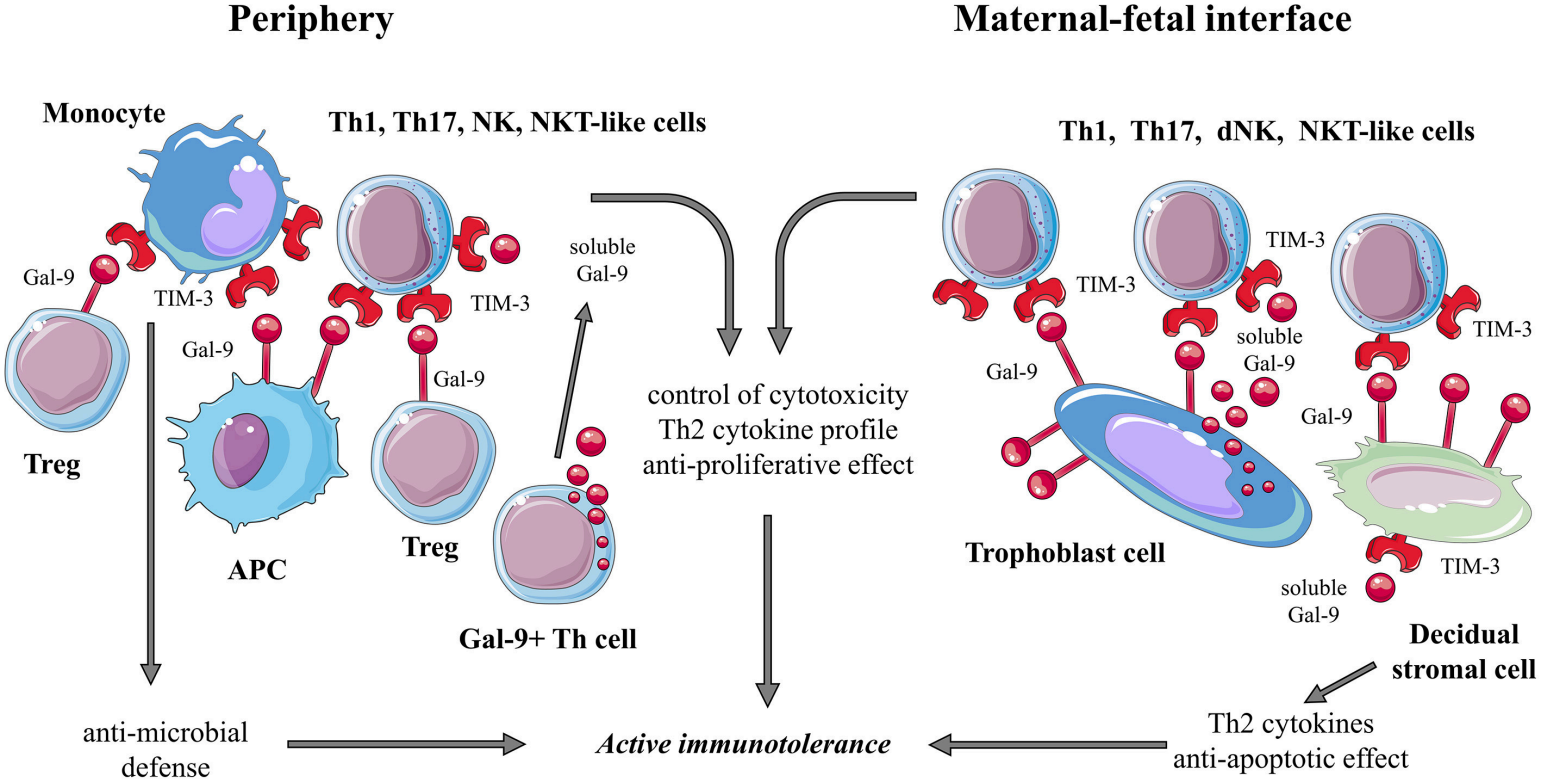
Summarizing the possible role of the TIM-3/Gal-9 pathway during human pregnancy.

TIM-3+ CD8+ T and NK cells show increased cytotoxicity in the third trimester of pregnancy suggesting altered functional capacities toward the end of pregnancy. The increasing levels of soluble Gal-9 throughout pregnancy might have a counter-regulatory function to control enhanced cytotoxicity of TIM-3+ CD8+ T and NK cells (57, 78). These data are inconsistent with other findings where TIM-3+ NK cells were found to have a high capacity to secrete Th2 type cytokines and reduced cytotoxicity toward trophoblast cells as a possible consequence of galectin-9/TIM-3 interaction (76).

TIM-3 expression on monocytes is regulated by IL-4 (upregulation) and IFN-γ (downregulation) cytokines, and it is involved in the effective anti-microbial immune defense by synergizing with TLR signaling (59).

It has been demonstrated, that TGF-β1 can induce peripheral NK cells to form decidual NK-like phenotype (79, 80). TGF-β1 treatment upregulated TIM-3 expression on peripheral NK cells proposing an important function of this co-receptor at the MFI (81).

#### TIM-3 at the Maternal-Fetal Interface

Although TIM-3 expression at the MFI was shown on different decidual lymphocyte subsets, like CD8+, CD4+ T cells, NK cells (69–71, 81), little is known about their role in successful implantation and placentation. The majority of decidual NK (dNK) cells express TIM-3 (60–90%) (69, 81). According to CD117/CD94 expression, TIM-3+ dNK cells have a mature phenotype with Th2 cytokine profile (69, 81). Secretion of IL-4 could be further increased and secretion of TNF-α could be decreased by recombinant human Gal-9 treatment of LPS stimulated dNK cells suggesting regulatory function of TIM-3+ dNK cells on the exaggerated inflammatory response since trophoblast is capable of secreting a large amount of Gal-9, as well as decidual tissue showed high galectin-9 expression (69, 81). Interestingly, blocking TIM-3 signal with TIM-3 fusion protein resulted in the reduction of IFN-γ and TNF-α production of dNK cells (81). Immunohistochemical studies demonstrated, that the fetal part of the MFI, trophoblast cells of term placenta highly express galectin-9 as well (78).

Beside decidual immune cells, decidual stromal cells (DSCs) also express TIM-3, and TIM-3+ DSCs produce higher levels of Th2 cytokines suggesting immune activities of the decidual tissue itself (82). Furthermore, TIM-3 activation seems to be anti-apoptotic when DSCs were stressed through Toll-like receptor activation which is a new potential of this molecule since it acts pro-apoptotic on CD4+ T cells (64, 82, 83).

#### TIM-3 in Pregnancy Complications

The possible involvement of the TIM-3/Galectin-9 pathway in the pathogenesis of unexplained miscarriages, recurrent spontaneous abortion (RSA), and preeclampsia (PE) has been studied by several groups, both in the periphery as well as at the MFI. However, data should be interpreted cautiously since the inclusion and exclusion criteria for these clinical syndromes and recruitment of the patients involved may vary.

In RSA patient, reduced TIM-3 expression level of peripheral NK cells was observed which could be the result of lower serum TGF-β1 levels, a lack of stimulus for upregulation of TIM-3 (76, 81). Besides TIM-3 surface expression changes, there is an increase of soluble TIM-3 (sTIM-3) and a decrease of soluble galectin-9 in the sera of these patients assuming enhanced competitive binding of galectin-9 by sTIM-3 leading to failed inhibitory signals controlling inflammation (76, 84). Furthermore, TIM-3+ NK cells of RSA patients produce more pro-inflammatory and less anti-inflammatory cytokines suggesting functional deficiencies (76). The only genetic polymorphism analysis of the TIM-3 gene was carried out in RSA patients. TIM-3 polymorphism can affect ligand binding properties and may be involved in some immune-mediated diseases (85). However, analyzing polymorphism of the promoter region of the TIM-3 gene, no differences between the different genotype frequencies could be observed in healthy pregnant women and RSA patients (86). At the MFI, immunohistochemical studies revealed reduced expression of TIM-3 by decidual tissue of women with RSA. Furthermore, same findings were confirmed in the case of DSCs by flow cytometry (82). A decreased percentage of TIM-3 by dNK cells was also demonstrated, although in patients with unexplained miscarriage not with RSA (69). Conflicting data exist according to decidual TIM-3 expression, one study found upregulated TIM-3 and galectin-9 expression in decidua and chorionic villi, both at mRNA and at the protein level in patients with RSA. The authors interpret these findings as being reactive to downregulate Th1 responses observed in RSA (87).

There are few inconsistent data about the possible role of the TIM-3/Gal-9 pathway in the pathogenesis of preeclampsia. On the one hand, in preeclampsia, both TIM-3 and Gal-9 were found to be upregulated in decidual tissue, and TIM-3 expression of peripheral blood monocytes was shown to increase (4). On the other hand, a decreased ratio of TIM+3+ Th, NK, and Vdelta2+ T cells could be confirmed in the peripheral blood of preeclamptic patients (48, 77). Both findings suggest disturbed immune regulation of Th1 responses due to altered Gal-9 and TIM-3 interactions.

## PD-1

PD-1 is a transmembrane receptor expressed by e.g., T cells, B cells, natural killer (NK) cells, antigen presenting cells (5, 88). PD-1 generates a strong inhibitory signal upon binding to its ligands PD-L1 and PD-L2, resulting in down-regulation of pro-inflammatory T-cell activity (89). PD-L1 can be found on several immune cells (resting T cells, B cells, dendritic cells, macrophages), in various tissues, like placenta, heart, spleen (5, 90, 91). In contrast to that, PD-L2 expression is limited to dendritic cells and macrophages (92). Furthermore, ligand expression of PD-1 can be regulated, e.g., through the local cytokine environment. PD-L1 expression is increased by many pro-inflammatory factors (LPS, GM-CSF, VEGF) and cytokines (IFN-γ, TNF-α) (5, 93, 94).

### PD-1 in Murine Pregnancy

In the allogeneic murine pregnancy model, surface expression of PD-1 on peripheral CD4+ and CD8+ T cells was not altered after conception and during gestation (1). PD-1 blockade *in vivo* was shown to enhance the proliferation of CD4+ and CD8+ T cells in unmated and pregnant mice and to erase the protective effect of Treg cells in Treg treated abortion-prone animals (1, 22).

There are only a few but very informative data about the local presence of PD-1 in murine pregnancy showing PD-1 expression by a broad spectrum of decidual lymphocyte subsets including CD4+ T cells, CD8+ T cells, T follicular helper cells, γδ T cells, NK, and NKT-like cells (1, 68, 95, 96). Furthermore, increased PD-1 expression by decidual NK, NKT-like, and γδ T cells was associated with the reduced cytolytic activity of these cells when compared to the periphery suggesting PD-1 dependent regulation of innate effector functions at the MFI (68).

Concerning the tissue distribution profile of the two ligands for PD-1, PD-L1, and PD-L2 at the MFI, both fetal and maternal compartments are involved: PD-L2 is expressed throughout the murine decidua, whereas PD-L1 expression is limited to the decidua basalis (97). Insufficient data exist about PD-L expression by the trophoblast suggesting PD-L1 expression by the syncytiotrophoblast but not by trophoblastic giant cells, which are next to the decidua basalis (97, 98). Therefore, PD-1 interaction with its ligands may occur in the decidua itself and is not affected by the fetal part. *In vivo* blockade of PD-1 ligands in allogenic murine pregnancy highlighted the functional role of the PD-1/PD-L1 pathway since anti-PD-L1 treatment resulted in increased fetal resorption rate and a reduction in the litter size, whereas PD-L2 blockade had no effect on fetal resorption (97). At the MFI, PD-L1 blockade resulted in infiltration of T cells, complement deposits, and higher levels of IFN-γ suggesting T cell-mediated rejection mechanisms locally. Another supportive report on the protective role of the PD-1/PD-L1 interaction in maternal-fetal tolerance was revealed by observations of the PD-L1 deficient pregnant mice, which showed similar results in fetal resorption rate, litter size, and a shift toward Th17 emphasizing the role of PD-L1 expressing regulatory T cells controlling fetal antigen-specific maternal T cell (99, 100). Yet, data are conflicting since in another experimental setting neither PD-L1 nor PD-1 deficient mice had significant alterations in gestational or in neonatal offspring parameters (101). These findings indicate doubt about the role of the PD-1/PD-L1 pathway in the survival of the fetal allograft in mice and further studies are needed to reconcile previous controversial results.

### PD-1 in Human Pregnancy (Figure 3)

#### PD-1 at the Periphery

Although immunological acceptance of the fetus is primarily based on maternal tolerance mechanisms at the MFI locally, it exerts a significant impact on systemic immunity as well (102). Despite the fact that syncytiotrophoblast cells—which are bathed in maternal blood—express PD-L1 and PD-L2, data about possible changes in the PD-1 mediated systemic immune response during human pregnancy compared to healthy, non-pregnant controls are lacking (90, 103). The only information regarding this topic is that the frequency of PD-1 expressing T lymphocytes is elevated in the blood of healthy pregnant women compared to non-pregnant counterparts and soluble PD-L1 levels increase throughout gestation (78).

**Figure 3:**
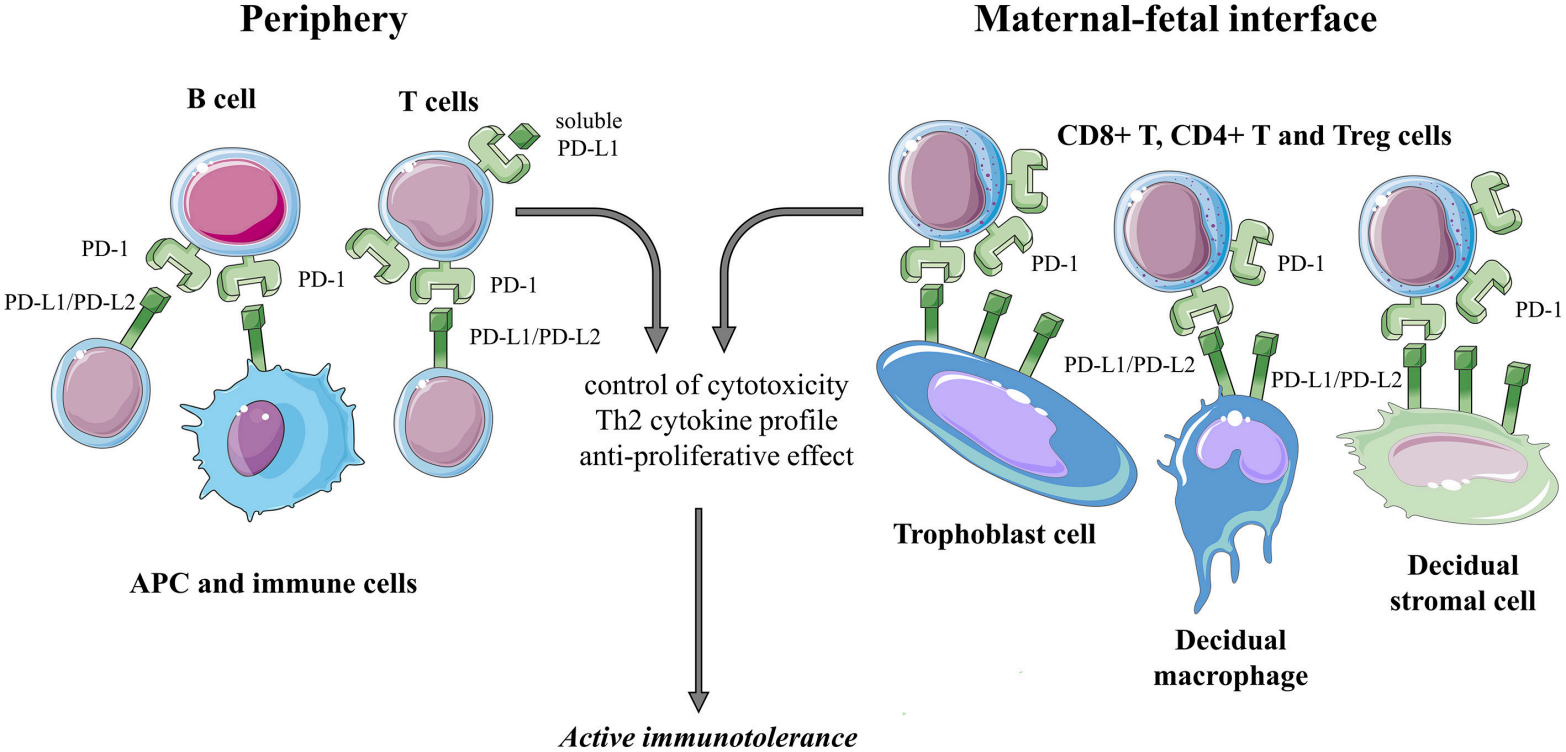
Summarizing the possible role of the PD-1/PD-L1, PD-L2 pathway during human pregnancy.

#### PD-1 at the Maternal-Fetal Interface

Immunofluorescent studies revealed a significantly higher PD-1 expression by decidual T lymphocytes similarly to non-pregnant endometrial T cells. This increase in PD-1 expression was demonstrated more in detail when compared to the periphery during pregnancy: decidual CD8+, CD4+, and regulatory T cells were shown to enhance PD-1 expression (104).

As already mentioned before, PD-L1 and PD-L2 are present in the placenta throughout pregnancy (90, 103, 105). In the first trimester, the major fetal source of PD-L1 is the villous syncytiotrophoblast and extravillous cytotrophoblast, while PD-L2 expression is much more restricted to villous cytotrophoblast (103, 105). On the maternal side, decidual stromal cells constitutively express both PD-L1 and PD-L2, but Th1 cytokines can further enhance their surface expression. PD-L1 expression by decidual macrophages is evident as well (104, 106).

PD-1 interaction with its ligand PD-L1 in different co-culture experiments resulted in reduced Th1 cytokine production by CD4+ T cells (104, 106). These findings suggest the contribution of the PD-1 mediated pathway to the establishment of a favorable Th2 type immune balance at the FMI in healthy human pregnancy.

#### PD-1 in Pregnancy Complications

Limited information is available about the involvement of the PD-1/PD-L pathway in pregnancy disorders. In preeclampsia, the percentage of PD-1 positive regulatory T cells was significantly higher than in healthy pregnancy with no difference in their PD-L1 expression. While the ratio of PD-1+ Th17 cells was not altered, the PDL1 expression by Th17 cells increased. PD-1 expression by CD3+, and CD4+ T cells did not significantly differ suggesting dysregulated PD-1/PD-L1 axis within the Treg/Th17 imbalance in the clinical phase of preeclampsia (47, 107).

In the case of RSA, decidual PD-L1 expression was significantly reduced on both mRNA as well as on protein level compared to healthy first-trimester decidua, while PD-1 expression by decidual lymphocytes showed no difference (108).

#### TIM-3 /PD-1 Co-expression

Effector cells of the immune system can be characterized by co-expression of different co-inhibitory molecules (109). In human pregnancy, TIM-3+PD-1+ CD8+ T cells preferentially accumulate in the decidua (71). Upregulation of both TIM-3 and PD-1 by decidual CD8+ T cells might be induced by embryonic trophoblast in an HLA-C dependent manner (71). These double positive cells display higher proliferative activity and produce more Th2 type cytokines than their TIM-3/PD-1 double negative CD8+ counterparts (71). Blocking both co-receptors increased cytotoxicity and decreased Th2 type cytokine production of TIM-3+PD-1+ CD8+ T cells suggesting a protective, anti-inflammatory role of TIM-3 and PD-1 co-expressing decidual CD8+ T lymphocytes at the MFI (71). This hypothesis is strengthened by further observations both in human as well as in mice. In murine pregnancy systemic blockade of both TIM-3 and PD-1 *in vivo* resulted in further reduction of fetal growth and litter size when compared to the blockade of either TIM-3 or PD-1 alone (71). In line with these findings, in patients with RSA, dual expression of TIM-3 and PD-1 by decidual CD8+ T cells was found to be significantly reduced and to be less proliferative in contrast to a healthy pregnancy (71).

## Summary

Immune checkpoint molecules have a major impact on cellular immunity by limiting inflammatory immune response and thereby maintaining physiologic tissue conditions. From this point of view, maternal-fetal immunotolerance represents a real immunological challenge for the immune system of the mother where accurate, tight, and dynamic immune control is required for healthy pregnancy proceeding from the time of implantation on.

As presented in this review in detail, the possible role of immune checkpoint molecules in the establishment of maternal-fetal immunotolerance has been extensively studied. In the case of CTLA-4, TIM-3, and PD-1, the participation and possible role of these molecules in maternal immune response have been confirmed by different approaches, e.g., animal and human experiments, *in vitro* and *in vivo* studies. Table 1 shows the different mice strains used in animal experiments. However, data are sometimes conflicting and not comprehensive, which may be due to experimental setting differences, small sample size, and the highly complex and multilevel characteristics of immune cell activation. When considering the involvement of co-activatory molecules in maternofetal immune interactions, the co-signaling network is far more complicated (110).

**Table 1:** Type of mouse models used in the experiments.

**Type of mouse models used in the experiments**	**References**
**NORMAL ALLOGENEIC PREGNANCY MATINGS**	
CBA/J × BALB/c	(16, 17, 20–22, 69–71)
CBA/J × C57BL/6	(66, 67, 97, 99, 100)
BALB/c × C57BL/6	(95, 96, 101)
**NORMAL SYNGENEIC PREGNANCY MATINGS**	
CBA/J × CBA/J	(67, 96)
BALB/c × BALB/c	(68)
C57BL/6 × C57BL/6	(1, 96, 101)
**ABORTION-PRONE ALLOGENEIC MATING**	
CBA/J × DBA/2	(15–17, 20–23, 69, 70)
**NON-PREGNANT MICE**	
BALB/c	(18, 19, 73, 98)
C57BL/6	(18, 19, 74, 75, 98)

Although there are no current clinical trials aiming at immune checkpoint molecules and interactions in pregnancy complications, some results of the studies discussed in this paper indicate the possible role of TIM-3 cell surface expression rate and serum levels of the soluble ligands PD-L1 and galectin-9 as potential biomarkers for screening during pregnancy (57, 76, 78).

Immune checkpoint inhibitors targeting the PD-1/PD-L1 and CTLA-4 pathways are revolutionary therapeutics in advanced malignancies and could be used in the treatment of chronic viral infections (HIV, HCV) as well in the future. Because of lacking a (68) adequate and well-controlled studies and based on the findings in mouse models, where blockade of the PD-1/PD-L1 pathway resulted in the adverse effect on pregnancy, checkpoint inhibitors are relatively contraindicated for the treatment of metastatic cancer in pregnant women requiring an individualized decision in each case (22, 97, 100, 111, 112).

Although intensive research and a large amount of information regarding the involvement of immune checkpoint molecules in reproductive immunology, the puzzle is not complete. Our current knowledge is quite deficient since there are several other immune checkpoint molecules described recently: Lymphocyte-activated gene-3 (LAG-3), T cell immunoreceptor with Ig and ITIM domains (TIGIT), B and T lymphocytes attenuator (BTLA), V-domain Ig suppressor of T cell activation (VISTA) are novel members of immune checkpoint molecules with proven immune regulatory activity (113). Until today, studies regarding this new generation of negative checkpoint regulators in the field of reproductive immunology are missing and urgently needed.

## Author Contributions

EM writing, original draft preparation. MM and AB original draft preparation. KD table and figure preparation and editing. LS writing, review, and editing.

### Conflict of Interest Statement

The authors declare that the research was conducted in the absence of any commercial or financial relationships that could be construed as a potential conflict of interest.
